# Genomic selection to resistance to *Stenocarpella maydis* in maize lines using DArTseq markers

**DOI:** 10.1186/s12863-016-0392-3

**Published:** 2016-06-18

**Authors:** Jhonathan Pedroso Rigal dos Santos, Luiz Paulo Miranda Pires, Renato Coelho de Castro Vasconcellos, Gabriela Santos Pereira, Renzo Garcia Von Pinho, Marcio Balestre

**Affiliations:** Department of Biology, Federal University of Lavras, Lavras, MG CP 3037 Brazil; Department of Agriculture, Federal University of Lavras, Lavras, MG CP 3037 Brazil; Department of Exact Science, Federal University of Lavras, Lavras, MG CP 3037 Brazil

**Keywords:** Ear rot, Genetic groups, Ridge regression best linear unbiased prediction, Bayesian stochastic search variable

## Abstract

**Background:**

The identification of lines resistant to ear diseases is of great importance in maize breeding because such diseases directly interfere with kernel quality and yield. Among these diseases, ear rot disease is widely relevant due to significant decrease in grain yield. Ear rot may be caused by the fungus *Stenocarpella maydi;* however, little information about genetic resistance to this pathogen is available in maize, mainly related to candidate genes in genome. In order to exploit this genome information we used 23.154 Dart-seq markers in 238 lines and apply genome-wide selection to select resistance genotypes. We divide the lines into clusters to identify groups related to resistance to *Stenocarpella maydi* and use Bayesian stochastic search variable approach and rr-BLUP methods to comparate their selection results.

**Results:**

Through a principal component analysis (PCA) and hierarchical clustering, it was observed that the three main genetic groups (Stiff Stalk Synthetic, Non-Stiff Stalk Synthetic and Tropical) were clustered in a consistent manner, and information on the resistance sources could be obtained according to the line of origin where populations derived from genetic subgroup Suwan presenting higher levels of resistance. The ridge regression best linear unbiased prediction (rr-BLUP) and Bayesian stochastic search variable (BSSV) models presented equivalent abilities regarding predictive processes.

**Conclusion:**

Our work showed that is possible to select maize lines presenting a high resistance to *Stenocarpella maydis*. This claim is based on the acceptable level of predictive accuracy obtained by Genome-wide Selection (GWS) using different models. Furthermore, the lines related to background Suwan present a higher level of resistance than lines related to other groups.

**Electronic supplementary material:**

The online version of this article (doi:10.1186/s12863-016-0392-3) contains supplementary material, which is available to authorized users.

## Background

Throughout its evolution, maize has undergone an intensive domestication process and concurrently it has presented particular susceptibility to certain pathogenic microorganisms that directly influence kernel production and quality, such as *Stenocarpella maydis*, which is a fungus responsible for rot in ears and kernels and causes a disease known as ear rot.

In addition, to losses in yield, the nutritional and economic values of the kernels may be depreciated because of mycotoxins known as diplodiatoxins, which may compromise the final feed quality and could be toxic to birds and cattle [26]. The association of the fungus *S. maydis* with corn seeds may also substantially compromise germination and seedling vigor [34].

The harmful economic impact of this disease increases every year and is driven by increases in the use of irrigated areas as well as by the use of no-tillage systems. These factors contribute to the propagation and survival of *S. maydis* in farming areas because of its necrotrophic nature. Moreover, ear rot occurs in both tropical and temperate regions; thus, it is a disease of global importance [4, 36].

Certain agronomic practices have been suggested to reduce *S. maydis* inoculum, such as crop rotation, sowing healthy seeds, planting at the recommended density, and using resistant cultivars [7]. According to these Casa et al. [7], crop rotation has been adopted because the microorganism can survive as a saprophyte in maize residue over harvest intervals of up to 320 days. The efficacy of chemical control of this disease is still debatable, although studies are showing an increase of up to 12 % in kernel yield upon implementation of this practice [6]. Among the strategies to control infestations of ear rot, genetically resistant plants are considered to be low-cost alternatives that have high effectiveness and no environmental impact [1].

Despite the clear advantage of plant breeding to obtain resistant genotypes, there is a considerable lack of resistant cultivars. Therefore, breeding programs from public and private institutions must work intensively to obtain cultivars resistant to *S. maydis*.

Plant breeding used to target resistance to ear rot is usually performed using traditional phenotypic analysis methods, with data obtained in studies conducted in environments with high disease pressure. Evaluations of this disease are performed via secondary traits that may be used effectively in the selection of plants resistant to *S. maydis*, such as in the percentage of rotten ears and cobs and tilting of ears in the plant [33]. In practice, mass phenotypic selection is generally applied in early generations, but this practice is not efficient, which is possible because of low trait heritability and high environment interactions [28].

In addition to phenotypic selection, the identification of quantitative trait loci (QTLs) and the application of marker-assisted selection (MAS) practices [36] are also common in breeding programs. The MAS, based on QTL mapping take into account the gene identification in disequilibrium with molecular markers in structured populations [2, 29]. However, despite initially high expectations, few highly relevant results have been obtained from the use of this technique [11, 20].

An efficient alternative to mitigating certain limitations of MAS was suggested by Meuwissen et al. [25]. The proposed method is popularly known as genomic selection (GS) and based on the massive use of molecular markers distributed throughout the genome. Because of the high level of linkage disequilibrium between the marker and QTL, this method does not require structured populations [11, 16].

The statistical models to be adopted in Genomic Selection (GS) greatly depend on the genetic architecture to be studied. In general, infinitesimal models, such as genomic and ridge regression best linear unbiased prediction (GBLUP and rrBLUP, respectively), have a good predictive power and can adequately describe the genetic architecture in infinitesimal models [19]. The infinitesimal model is widely accepted in quantitative genetics, although its application in molecular genetics is still very discussed, and although several genes have been observed, the infinitesimal assumption may still be strong [14, 24]. The infinitesimal assumption claims that individual genotype is based on the sum of infinitesimal independent locus acting additively on the trait and presenting Gaussian properties; therefore, it is founded in the central limit theorem. Thus, Bayesian models may be more efficient for describing the genetic architecture when several (but don’t infinitesimal) genes control the trait because they present a polygenic profile and high resolution in the identification of large-effect genes [11, 15].

Because of the scarcity of information available on the genetic mechanisms of resistance to ear rot and lack of studies to identify genomic regions involved in resistance to *S. maydis,* the objectives of this work are: (i) evaluate the usefulness of GS in the selection of genotypes resistant to *S. maydis;* (ii) compare the rrBLUP and Bayesian stochastic search variable (BSSV) selection methods in terms of the selection (iv) genetically characterize the germplasm bank of the Federal University of Lavras for resistance to ear rot.

## Results

### Genetic germplasm characterization through a principal component analysis

**Fig. 1:**
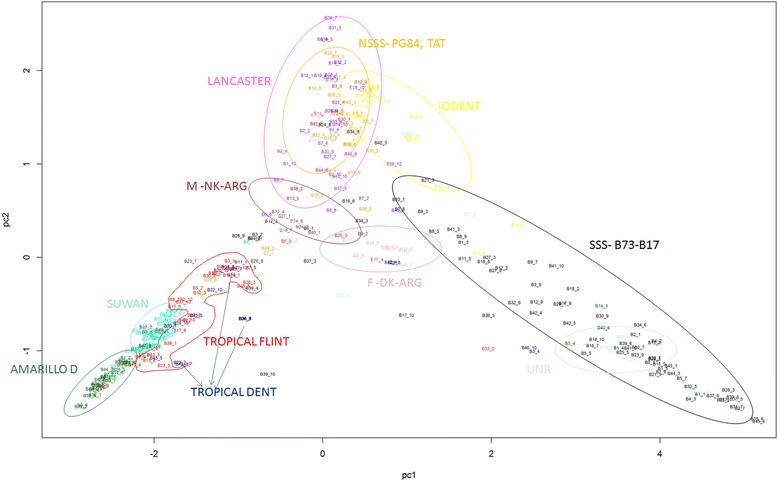
Genetic clustering of inbred lines from the germplasm bank of the Department of Agriculture of UFLA using a principal component analysis

The genomic relationships among the lines obtained by 23,154 Dart-seq markers were submitted to spectral decomposition. In total, it was observed 6 % of missing data point and it were imputed using the EM approach by A.mat function in rr-BLUP library deleting markers presenting more than 90 % of missing data. The inbred lines were clustered into distinct genetic groups through a Principal Component Analysis (PCA) analysis based on the relationship data. This approach was effective in the clustering of our genetic background even explained just 15.24 % of the genomic additive matrix (Fig. 1).

**Fig. 2:**
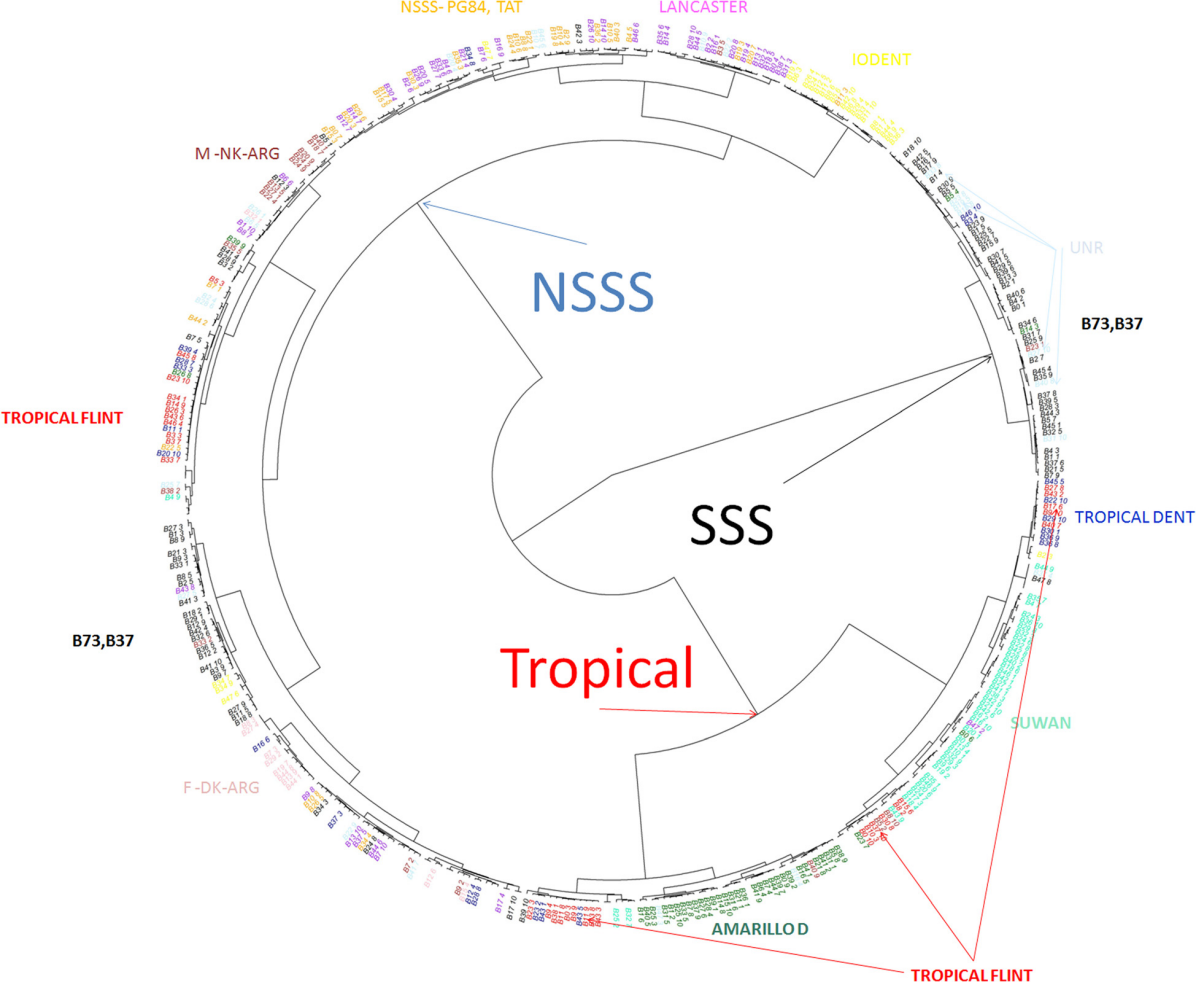
Hierarchical clustering of the inbred lines from the germplasm bank of the Department of Agriculture of UFLA using the Euclidean distance of the elements of relationship matrix **A**

A clear distinction between the tropical genetic subgroups Suwan, Amarillo Dent, Tropical Flint and Tropical Dent may be observed in the left lower corner of Fig. 1. The colors reflect the empirical knowledge of the breeder about the germplasm and its position after clustering. This figure clearly shows a pyramid-shaped cluster, which includes the three most important groups used in the breeding program. The somewhat overlapping temperate subgroups Iodent, Lancaster, Non-Stiff Stalk Synthetic (NSSS), NSS-PG84, M-NK-ARG and F-DK-ARG are highlighted in the upper vertex of the pyramid and grouped separately from the lines of tropical origin. The Stiff Stalk Synthetic (SSS) group was derived from crosses of lines B73 and B17 with other lines and allocated in the right lower vertex of the biplot, thus representing a cluster distant from the lines of temperate origin, which was expected because of the known high heterotic pattern between these two genetic groups. In the center, it is possible to observe the genetic group F-DK-ARG, and this spatial pattern suggests that the lines belonging to this group were derived from a pool between temperate germplasm. The genetic groups defined by the hierarchical clustering method confirm the results obtained in the PCA analysis (Fig. 2). For example, subgroups Amarillo Dent, Tropical Flint and Suwan, which were clustered in the tropical genetic group in the PCA, were also similarly differentiated from the NSSS and SSS groups by the hierarchical clustering technique.

### Genomic prediction and comparison between ERIS, PRK and NESR

Among the disease evaluation methods used here, markers with a non-null effect were not observed for the number of ears with symptoms of rot (NESR) trait; thus, when the BSSV approach was used, the *ρ* mixing parameter was estimated with a probability close to one. Based on the sample information, this result suggests that the probability of identifying genes with effects different from zero is negligible for this trait.

For the percentage of rotten kernels (PRK) and Ear Rot Incidence Score (ERIS) traits, the *ρ* mixing parameter values of the BSSV model were 0.37 and 0.32, respectively. This result suggests the presence of sufficient sample information for the identification of genes with probabilities different from zero. Therefore, cross-validation analyses were performed for PRK and ERIS only using the rrBLUP and BSSV methods.

As shown in Table 1, using the PRK trait provided clear advantages for both the rrBLUP and BSSV methods compared with the ERIS.

**Table 1:** Model performance based on coefficient of determination between individual predicted genomic breeding values and observed breeding values obtained through cross-validation using the rrBLUP and BSSV methods by phenotyping per proportion of rotten kernels (PRK) and ear rot incidence score (ERIS)

		${r}_{{}_{\left({\widehat{\mathbf{y}}}_p,\kern0.5em Z\widehat{a}\right)}}^2$ (T1)	${r}_{{}_{\left({\widehat{\mathbf{y}}}_p,\kern0.5em Z\widehat{a}\right)}}^2$ (T2)	${r}_{{}_{\left({\widehat{\mathbf{y}}}_p,\kern0.5em Z\widehat{a}\right)}}^2$ (T3)	${r}_{{}_{\left({\widehat{\mathbf{y}}}_p,\kern0.5em Z\widehat{a}\right)}}^2$ (T4)	${r}_{{}_{\left({\widehat{\mathbf{y}}}_p,\kern0.5em Z\widehat{a}\right)}}^2$ (T5)	Mean
PRK	rrBLUP	0.846	0.767	0.905	0.931	0.900	0.878 (0.065)
	BSSV	0.887	0.804	0.835	0.950	0.893	0.874 (0.056)
ERIS	rrBLUP	0.861	0.664	0.588	0.759	0.627	0.699 (0.110)
	BSSV	0.729	0.764	0.664	0.629	0.680	0.693 (0.053)

${r}_{{}_{\left({\widehat{\mathbf{y}}}_p,\kern0.5em \mathbf{Z}\widehat{\mathbf{a}}\right)}}^2$ coefficient of determination between the predicted breeding value *ŷ*_*p*_ and observed breeding value *Za* obtained in the cross-validation; T1, T2, T3, T4 and T5 are the training populations 1, 2, 3, 4 and 5, respectively. The values between parenthesis represent the standard deviations

The PRK trait provides a higher predictive power when we use the predicted breeding values obtained from the training population (*n-k*) and the observed breeding value from full data (*n)* i.e., r^2^ = 0.878 and r^2^ = 0.874 for rr-BLUP and SSVS respectively. That is approximately 10.84 % (rrBLUP) and 10.96 % (BSSV) higher compared with the ERIS trait. On the other hand, when we used the predictive ability as a measure of accuracy based on phenotypic values, it was not so high, ranging from 0.241 to 0.569 (Table 2). It was roughly 31 % lower than predictions based on breeding value for PKH and 40 % for ERIS. This difference in prediction is because the phenotypic values include residual variance and in this situations the accuracy threshold is linked to the heritability (h^2^ = 0.648 for PKH and 0.265 for ERIS). In general, the rr-BLUP and BSSV were equivalent in the prediction of the Genomic Breeding Values GBVs for both traits.

**Table 2:** Model performance based on coefficient of determination between individual predicted genomic breeding values and observed phenotype obtained through cross-validation using the rrBLUP and BSSV methods by phenotyping per proportion of rotten kernels (PRK) and ear rot incidence score (ERIS)

		${r}_{{}_{\left({\widehat{\mathbf{y}}}_p,\kern0.5em \mathbf{y}\right)}}^2$ (T1)	${r}_{{}_{\left({\widehat{\mathbf{y}}}_p,\kern0.5em \mathbf{y}\right)}}^2$ (T2)	${r}_{{}_{\left({\widehat{\mathbf{y}}}_p,\kern0.5em \mathbf{y}\right)}}^2$ (T3)	${r}_{{}_{\left({\widehat{\mathbf{y}}}_p,\kern0.5em \mathbf{y}\right)}}^2$ (T4)	${r}_{{}_{\left({\widehat{\mathbf{y}}}_p,\kern0.5em \mathbf{y}\right)}}^2$ (T5)	Mean
PRK	rrBLUP	0.544	0.594	0.497	0.621	0.587	0.569 (0.048)
	BSSV	0.547	0.614	0.504	0.583	0.594	0.568 (0.043)
ERIS	rrBLUP	0.265	0.257	0.197	0.245	0.241	0.241 (0.030)
	BSSV	0.263	0.200	0.281	0.258	0.24	0.248 (0.035)

${r}_{{}_{\left({\widehat{\mathbf{y}}}_p,\kern0.5em \mathbf{y}\right)}}^2$ coefficient of determination between the predicted breeding value *ŷ*_*p*_ and observed phenotypic values ***y*** obtained in the cross-validation; T1, T2, T3, T4 and T5 are the training populations 1, 2, 3, 4 and 5, respectively. The values between parenthesis represent the standard deviations

This result suggests that GBVs may be predicted with high accuracy for the selection of lines resistant to *S. maydis* but, only a moderate accuracy was obtained in the prediction of phenotypic values*.*

### Germplasm sources of resistance to *S. maydis*

Following the genomic analysis and calculation of GBVs via rrBLUP and BSSV, 10 % of the most resistant and susceptible inbred lines were classified based on predicted values. The highest proportion of lines resistant to *S. maydis* was allocated to the Suwan genetic group for both methods (Table 3). For the BSSV method, 62.50 % of the inbred lines were concentrated in the Suwan group, whereas for rrBLUP, this proportion was 79.17 %. These results suggest that the Suwan genetic subgroup concentrates a higher proportion of germplasm with alleles favorable to resistance to ear rot. As reported by Rossouw et al. [33], the majority of germplasm resistant to *S. maydis* is of tropical origin, which corroborates our results because the Suwan genetic group (belonging to the tropical group) was identified as the largest source of resistance to this pathogen. In the class of susceptible lines, the methods produced conflicting results regarding the genetic group with the highest proportion of these genotypes. The predominantly susceptible group identified by the BSSV analysis was SSS (25 %), whereas the predominant group identified by rrBLUP was IODENT (29.17 %).

**Table 3:** Classification of 10 % of the most susceptible (S) and resistant (R) inbred lines for the trait proportion of rotten kernels (PRK) in the 13 genetic groups defined by the principal component analysis (PCA) for the rrBLUP and BSSV models

Groups	rrBLUP		BSSV	
	S	R	S	R
Mixed	12.50 %	4.17 %	12.50 %	4.17 %
Amarillo Dent	0 %	8.33 %	4.17 %	16.67 %
Female DK	0 %	0 %	0 %	4.17 %
Female UNR Temperate	0 %	0 %	8.33 %	0 %
IODENT	29.17 %	0 %	20.83 %	0 %
Lancaster	25.00 %	4.17 %	16.67 %	4.17 %
Male Temperate	0 %	4.17 %	4.17 %	0 %
Non-Stiff Stalk	4.17 %	0 %	8.33 %	0 %
SSS	25.00 %	0 %	25.00 %	0 %
Suwan	0 %	79.17 %	0 %	62.50 %
Temperate Dent	0 %	0 %	0 %	0 %
Tropical Flint	0 %	0 %	0 %	0 %
UNR	4.17 %	0 %	0 %	4.17 %

## Discussion

Disease evaluation and line selection in tropical environments constitutes a great challenge for breeders. In addition to the difficulty of obtaining reliable methods, interactions between genotypes and environments further hamper the selection of superior genotypes. In this work, a high Genotype –By- Environment (G x E) interaction was observed in the analysis of phenotyping data from ERIS, PRK and NESR. In the preliminary data analysis obtained with these methods, genetic variances of 67,843, 0.04709 and 18.2104 were observed, whereas the genetic variances of the G x E component were 12,921, 0.4766 and 7.1249 respectively. This strong interaction may have been caused by differences in climate between the two environments. The climate in Lavras is classified as highland tropical, whereas the climate of Uberlândia is classified as tropical with a dry season. The incidence of this pathogen is generally restricted to higher altitudes and humidity environments, which include the region of Lavras. Thus, we believe that this difference in climates may be the factor that triggered the high G x E interaction observed between those two environments.

Regarding the method of evaluating pathogen incidence, our results indicated that evaluating resistance to ear rot is problematic. In general, the ERIS and NESR measures presented low heritability compared with PKR, although these three measures were highly correlated in the lines, with the NESR and PRK traits showing a correlation of 0.92. Thus, we suggest that the PRK trait may be used as a parameter in the evaluation of resistance to *S. maydis* because it shows higher heritability and is highly correlated with direct measures of disease incidence, such as ERIS and NESR. Moreover, unlike ERIS, the PRK method is not a subjective method. The quantitative nature of PRK resulted in improved predictions and identification of regions of resistance to *S. maydis*. It is worth noting that these three measures correlated positively among themselves and negatively with weight of ears without husk. For example, the correlation between NESR quantified by the weight of ears without husk −0.84 [28], which suggests that the selection of lines with heavier ears contributes to more resistant genotypes.

As shown in our study, selecting the model that best describes the genetic architecture may be decisive when adopting a breeding strategy, such as when a breeder only wants to select the most resistant lines or perform MAS. Thus, the results of cross-validating and identifying candidate genes may aid the breeder during decision making.

In the cross-validation process using the BSSV and rrBLUP methods, differences were not observed between both procedures for GBV prediction and only a slight difference for phenotypic value predictions (Tables 1 and 2). These results corroborate studies that compared direct regression models in the genome [10, 21, 23] and suggest that differences in prediction power are marginal and attenuated with the cross-validation procedure [11, 17].

In regular studies involving GWS and cross-validation methods, the supervised learning process is applied to evaluate the model performance based on its prediction ability for missing data. It is very usual to use the correlation between predicted GBV and phenotype values where the residual is assumed as a nuisance amount. In this scenario, the maximal correlation is limited by the trait heritability. On the other hand, if the residuals are removed from the phenotypic values, the GBV might be assumed as “true” values, where the squared correlation threshold is equal to 1. It is because the covariance among missing genotypes is equal to their variance (see Methods). In this study, the difference between the two accuracies measures is evident, and given that the residual is a spurious amount in genetic improvement, we could suggest the use of the correlation between the GBVs instead the GBVs vs. phenotypic values. However, we agree that this suggestion is useful only in cross-validation and statistical context; in practice, the prediction of phenotypic values may present a better view of real genome-wide selection efficiency.

Another important point about the cross-validation is related to the necessity of performing repeated k-fold to evaluate the reliability of the prediction measure (*r*^2^). Wray et al. [38] discuss the aspects of independence between training and validation dataset under fixed GWAS models. Baumann and Baumann [3] compare some repeated cross-validations approaches and show that shrinkage models such as LASSO are less influenced by the cross-validation bias. In our work, both models are taken as shrinkage models and given that our Bayesian approach demands high computational effort it is very costly to perform repeated cross-validation under MCMC models such as SSVS. However, we observed that for the rr-BLUP based on mixed models, the running mean obtained across 100 rounds of 5-fold cross-validation were very close to showed in Tables 1 and 2 (Additional file 1: Figure S1).

As indicated by Habier et al. [18], despite the models used in GS having a similar predictive power, there are variations in the methods by which genetic information is retrieved. For example, Habier et al. [18] suggested that the rrBLUP method (which represents an infinitesimal model) tends to more efficiently capture genetic relationship information, whereas the BayesB model (polygenic model of specific variance) tends to retrieve primarily information on QTL-marker linkage disequilibrium. The BSSV method as presented in this work is a (conceptually) polygenic method, and unlike BayesB, the mixing proportion is a Bernoulli random variable [25].

The identity by state (IBS) analysis obtained by the line markers matrix showed a pyramidal cluster of heterotic groups in our breeding program. The separation of tropical groups, SSS and NSSS was evident with both clustering methods.

The PCA-based cluster analysis strategies of maize inbred lines were performed in a similar way by Romay et al. [32], who characterized 2815 inbred maize lines belonging to the germplasm bank of the US Department of Agriculture (USDA) using the genotyping-by-sequencing (GBS) technique with 681,257 SNPs. Despite the high density of the marker panel and large number of evaluated lines, consistent clustering was not observed among the genetic groups, which may have been caused by the exclusion of the unified relationship matrix *A* as a source of information for the spectral decomposition because these authors used the markers’ Euclidean distance matrix. [22] argue that the population structure can be retrieved in the first principal components in PCA while high-order components represent the kinship among the individual. This claim could explain why or PCA analysis was able of separating the population structure even explaining only 15.24 % of the additive matrix.

In the clustering pattern obtained by Romay et al. [32], strong overlapping occurs between the genetic groups, whereas a clear distinction between groups was obtained with our strategy. To confirm our hypothesis, the data used by Romay et al. were subjected to the new analysis, and a cross-shaped pattern was observed for these same data (unpublished data).

Because of the adequate group characterization, most of the resistance sources (almost 80 %) are clustered in the tropical material as expected. Also, the lines belonging to the SSS and IODENT group of temperate origin were the most susceptible. This result, although expected, clarifies the importance of good germplasm characterization for a better understanding of resistance sources. The technique associated with GWAS and the identification of candidate genes regions provides breeders with a powerful tool in the selection process. We must note that the inheritance of resistance to *S. maydis*, such as dominance and epistasis effects, was not explored in depth in this work. Nonetheless, our results are a starting point for improving the introduction of resistance alleles in susceptible lines and for performing directed crosses.

## Conclusions

Our results showed that the PRK trait may be used as an evaluation method in the genomic selection and for resistance to *S. maydis*. The rrBLUP and BSSV methods present the same efficiency in the prediction of resistant lines. In addition, the use of a PCA along with additive relationship information was efficient at defining genetic groups. Thus, it was possible to identify groups resistant to *S. maydis* in tropical accessions, particularly in lines distributed within the Suwan genetic group.

## Methods

### Genetic characterization of the germplasm bank

Four hundred and forty-seven lines were genotyped with 23,154 DArTSeq™ obtained by Diversity Arrays Technology Pty. Ltd Yarralumia ACT, Australia. This technology is based on a complexity reduction method in order to obtain genome sequences copies and further sequencing based on next-generation sequencing using HiSeq2000 (Illumina, USA) More details about the method can be obtained in Raman et al. [30].

Missing data were imputed using the *A.mat* function and *mean* method in the rrBLUP package [12] of R software. Genomic relationships were calculated using the additive relationship matrix (**A**) proposed by Vitezica et al. [37] given by:

$$ \mathbf{A}=\frac{{\mathbf{W}}_{\mathbf{A}}{\mathbf{W}}_{\mathbf{A}}^{\hbox{'}}}{2\sum \mathbf{p}\mathbf{q}} $$

in which p is the frequency of the favorable allele; **q** is the frequency of the unfavorable allele; **W**_**A**_ is the deviation matrix of the markers centered in **p** (mean of the favorable allele for a given locus); and 2∑**pq** is the sum of the variances of the loci.

Genetic clustering of the inbred lines was performed by spectral decomposition of the relationship matrix **A**, and the first two principal components were subsequently plotted. Thus, instead to carry out the SVD from original genomic marker matrix we used the spectral decomposition of Vitezica et al. [37] positive definite matrix; to be more exact, since it is a square matrix we can use A = ULLU and subsequently one can apply the transformation A = ULV. After obtaining the plot, the consistency between the genetic cluster obtained with the markers and the known background was determined.

A hierarchical cluster analysis through the *hclust* function of the *hclust* package in R software [35] calculated by the *Wald* method was also conducted using a Euclidean distance matrix of the elements of the matrix *A* as an object.

### Field experiments and genotyping

The incidence of ear rot was evaluated in 238 lines of the 447 genotyped lines, together with four resistant controls from the germplasm bank of the Federal University of Lavras (Universidade Federal de Lavras - UFLA). Only elite lines were phenotyped while the others 209 were not since these lines were recently introduced in our breeding program and present a small number of evaluations. Therefore, the genome data for these lines were inserted in this study in order to present the pattern of our breeding program. The 238 lines were evaluated in crop year 2012/2013 in two environments in the municipalities of Lavras (910 m, 21°14’S and 45°00’W) and Uberlândia (863 m, 18°55’S and 48°16’) in the state of Minas Gerais, Brazil.

The population was evaluated in an augmented incomplete block design interspersed with common controls. The block consisted of 10 treatments (8 regular treatments and 2 common) and 3 replicates. The common treats are resistance and susceptive lines for *S. maydis*. The experimental plots consisted of a 3-m row with 0.7-m spacing.

### Pathogen culture, inoculation and evaluation

*S. maydis* isolates were obtained and replicated at the Seed Phytopathology Laboratory of the UFLA using the methodology by Clements et al. [9] with several modifications.

The isolates were cultured in complete medium for 30 days. After this period, the conidial suspension was adjusted using a Neubauer counting chamber to 10^6^ conidia*mL^−1^ on the day of the inoculation. Pathogen inoculation was performed 15 days after 100 % of the field plants had emitted the style-stigma using a pipette for the inoculation of 1 mL of isolate suspension into each corn ear.

The incidence of ear rot was evaluated based on three methods: (i) ear rot incidence score (ERIS); (ii) number of ears with symptoms of rot (NESR); and (iii) percentage of rotten kernels (PRK). A diagrammatic rating scale proposed by Reid et al. [31] was used in the ERIS evaluation method. The values of this scale range from 1 to 7 and included the following percentage severity categories: 1 (0 %); 2 (1-3 %); 3 (4-10 %); 4 (11-25 %); 5 (26-50 %); 6 (51-75 %); and 7 (76-100 %). The NESR was calculated as the number of ears that presented the characteristic symptoms of the disease relative to the total number of ears in the field. For the PRK, the evaluation was conducted according to the procedure proposed in decree no. 11 of 04/12/96 [5], which established a sample of 230 g of kernels per plot for visual separation and determination of the percentage of kernels showing discoloration in more than a fourth of the total surface.

### Data statistical analysis

Data analyses were performed in two stages. In the first phase, a mixed model was used for observation corrections according to the following effects: replicates, environments, genotypes *x* environments interaction (G X E) and residuals. The mixed model adopted was as follows:

1 $$ \mathbf{y}=\mathbf{X}\boldsymbol{\upbeta } +\mathbf{Tg}+\boldsymbol{\Omega} \mathbf{b}+\mathbf{W}\boldsymbol{\updelta } +\mathbf{e} $$

where *y* is the *n × 1* vector of observations; **X** is a *n × p* fixed effects incidence matrix (replication within local plus local); **T** is a *n × q* genetic effects incidence matrix; **Ω** is a random block effects incidence matrix within replicates; **W** is a line x environment interaction effects incidence matrix; and **β**, **g**, **b**, **δ** are vectors of the effects related to **X**, **T**, **Ω** and *W*, respectively and **e** represents the residual effects. The distribution of effects **g**, **b**, **δ** and **e** are assumed to be **N(0,** **σ**_**g**_^**2**^**)**, **N(0,** **σ**_**b**_^**2**^**)**, **N(0,** **σ**_**δ**_^**2**^**)** and **N(0,** **σ**_**e**_^**2**^**)**, respectively. The estimates of the best linear unbiased predictor (e-BLUPs) and variance components were obtained using residual maximum likelihood (REML) function maximization [27].

### Genomic analysis using the mixed models

The mixed model utilized in this study was calculated as follows:

2 $$ \overset{\smile }{\mathbf{y}}=\mathbf{j}\mathbf{u}+\mathbf{Z}\mathbf{a}+\mathbf{e} $$

where $\overset{\smile }{y}$ is a vector of the corrected means based on model 1, *n × 1*; **j** is a unit vector corresponding to the mean; **u** is the sample mean; **Z** is the marker’s genotype incidence matrix; and **a** and **e** are vectors of the additive genetic for each marker and residual effects, respectively.

The matrix of phenotypic variances **V** is given as follows:

$$ \mathbf{v}\mathbf{a}\mathbf{r}\left(\mathbf{y}\right)=\mathbf{Z}\mathbf{G}\mathbf{Z}\mathbf{\hbox{'}}+\mathbf{I}{\boldsymbol{\upsigma}}_{\mathbf{e}}^{\mathbf{2}}={\boldsymbol{\upsigma}}_{\mathbf{e}}^{\mathbf{2}}\left(\mathbf{K}\boldsymbol{\uplambda } +\mathbf{I}\right) $$

where **G** = **Aσ**_**a**_^**2**^ is an additive genetic variance matrix and **Iσ**_**e**_^**2**^ is the residual variance diagonal matrix and $\lambda =\frac{\sigma_a^2}{\sigma_e^2}$.

The GWAS analysis was performed with mixed.solve in the rrBLUP package [12] of R software.

### BSSV model

Among the Bayesian models proposed in the literature, the BSSV model was used in this study because of its ability to select large-effect markers in models with multiple markers. Adjustments to the original model proposed by Yi et al. [39] were proposed, and a new approach was used in order to encompass all marker effects and the model is calculated as follows:

3 $$ \overset{\smile }{y}=\mu +{\displaystyle {\sum}_{j=1}^m{z}_j{a}_j+e} $$

where $\overset{\smile }{y}$ is a vector of the corrected means based on model 1 *i* obtained by model 1, *μ* is the sample intercept, *z*_*ij*_ is the genotype of marker *j* of individual *i*, *a*_*j*_ is the effect of the marker *j* and *e*_*i*_ is the error of observation *i* following distribution **N(0,** **σ**_**e**_^**2**^**)**.

The acceptance of a marker effect depends on a combination of priori assumptions conditioned to a set of latent or indicator variables. Therefore, we can assume that the a priori additive effects of the markers are as follows:

$$ {a}_j\Big|\rho, {\varDelta}_j,\delta \sim \left(1-\rho \right)N\left(0,{\sigma_a^2}_j\right)+\rho N\left(0,\delta \right),j=1,\dots, K $$

where *σ*_*a*_^2^_*j*_ and *δ* represent high and low magnitude variance in the genetic marker effects, respectively. In this study, it was assumed a priori that

$$ {\sigma_a^2}_j\Big|a,b\sim inverse- escaled-{\chi}^2\left(v=4,{s}^2=0.002\right) $$

and *δ* = 10^− 6^ The prior hyperparameters *v* and *s*^2^ are related to Bayes A method described in [13]. The *δ* = 10^− 6^ corresponds to individual marker heritability at 1 % of phenotypic variance i,e *δ* = *σ*_*y*_^2^ × 0.01/*m*.

Another modification in the original BSSV method was the assumption that hyperparameter *ρ* was modeled in advance by a *Beta* distribution *ρ*|*a*, *b* ~ *Beta*(*a* = 1, *b* = 1) instead of 0.5 as originally described by Yi et al. [39]. The a priori distribution for the effects of the population mean was assumed to be constant, and the same distribution of *Δ*_*j*_ was assumed for residual variance *σ*_*e*_^2^.

The numerical integration of the posterior conditionals distribution was performed using the Markov chain Monte Carlo algorithm via Gibbs sampling [8], which is described by the following steps:

1. Sample *μ* of the full posterior conditional distribution:

$$ p\left(\mu \Big|....\right)\sim N\left[{\displaystyle \sum_{i=1}^n\left({y}_i-{\displaystyle \sum_{j=1}^k{z}_{ij}{a}_j}\right)/n,\frac{\sigma_e^2}{n}}\right] $$

2. Sample *a*_*j*_ of the full posterior conditional distribution:

$$ p\left({a}_j\Big|....\right)\sim N\left[{\left({\displaystyle \sum_{i=i}^n{z}_{ij}^2+\frac{\sigma_e^2}{v_{ai}}}\right)}^{-1}{\displaystyle \sum_{i=1}^n{z}_{ij}\left({y}_i-\mu -{\displaystyle \sum_{j\acute{\mkern6mu}\ne j}^k{z}_{ij\acute{\mkern6mu}}{a}_{j\acute{\mkern6mu}}}\right),{\left({\displaystyle \sum_{i=i}^n{z}_{ij}^2+\frac{\sigma_e^2}{v_k}}\right)}^{-1}{\sigma}_e^2}\right] $$

where *v*_*ai*_ = *η*_*i*_*σ*_*a*_^2^_*i*_ + (1 − *η*_*i*_)*δ*, with *η* : {1, 0} and *p*(*η*) ~ *Bernoulli*(*ρ*)

3. Sample ${\sigma}_{a_i}^2$ of an inverse chi-square distribution with the following parameters:

$$ p\left({\sigma}_{a_i}^2\Big|\dots \right)\sim inverse- escaled-{\chi}^2\left(v+1,{a}_i^2+v{s}^2\right) $$

4. Sample *η* of a Bernoulli distribution:

$$ p\left({\eta}_i=1\Big|....\right)\sim \frac{\rho N\left({a}_i\Big|0,{\sigma}_{a_i}^2\right)}{\rho N\left({a}_i\Big|0,{\sigma}_{a_i}^2\right)+\left(1-\rho \right)N\left({a}_j\Big|0,\delta \right)} $$

5. Sample *ρ*_*j*_ of a *Beta* distribution using the following conditional:

$$ p\left(\rho \Big|....\right)\sim Beta\left[1+{\displaystyle \sum_{j=1}^k{\eta}_k},1+\left(k-{\displaystyle \sum_{j=1}^k{\eta}_k}\right)\right] $$

6. Sample residual variance:

$$ p\left({\sigma}_e^2\Big|\dots \right)\sim inverse- escaled-{\chi}^2\left(v+n,RSS+v{s}^2\right) $$

where RSS is the residual sum of squares.

7. Repeat the steps described until convergence is attained.

The significance of the marker effects was determined with the *Wald test*. The statistics of this test *W*(*λ*) under the null hypothesis follow an asymptotic distribution *χ*^2^ with one degree of freedom. The test values may be obtained with

$$ W\left(\lambda \right)=\frac{a_{{}_j}^2}{\sigma_{aj}^2} $$

where ${\sigma}_{aj}^2={\left({\displaystyle \sum_{i=i}^n{z}_{ij}^2+\frac{\sigma_e^2}{v_{ai}}}\right)}^{-1}{\sigma}_e^2$. The critical value for marker acceptance was given by (*χ*_*tab*_^2^ = 3.84), considering an error rate of 5 %. The data set and the R program are available in Additional file 2 and Additional file 3 respectively.

### Cross-validation and correlations

The 5-Fold cross-validation method was used to assess the accuracy of the models. The set of 242 observations was randomly subdivided into five training populations, with four groups each containing 48 observations and one group containing 50 observations. One group was sequentially eliminated in the analysis process to be used as the validation population, and the remaining four groups were used as training populations *(n-k)* until all groups were used as the validation population. Predictions of the breeding values of lines (*ŷ*_*p*_) containing the validation population were based on

$$ {\widehat{y}}_{p_{(k)}}={Z}_ka $$

where *Z*_*k*_ is the marker matrix of the individuals belonging to the k-th validation population and *a* is the vector of the marker effects estimated for individuals from the training population.

The efficiency of prediction was measured by the determination coefficient (**r**^2^) between the predicted breeding values from validation set $$ {\widehat{\mathbf{y}}}_{{\mathbf{p}}_{\left(\mathbf{k}\right)}} $$ and the breeding values observed from full data analysis based on **Za**. In addition, the (**r**^2^) between $$ {\widehat{\mathbf{y}}}_{{\mathbf{p}}_{\left(\mathbf{k}\right)}} $$ and $$ \overset{\smile }{y} $$ (the corrected phenotypic values) was used to rescale the correlation to heritability threshold since this last measure takes into account the residual and genetic variances while the first approach based of BLUPs uses only the genetic variance. In other words, assuming $$ \operatorname{var}\left({\widehat{y}}_{p_{(k)}}\right)={\sigma}_a^2 $$$$ \operatorname{var}\left(\overset{\smile }{y}\right)={\sigma}_a^2+{\sigma}_e^2 $$ and assuming independence among $$ {\widehat{y}}_{p_{(k)}} $$ and the residuals *e*, the expected maximal squared Pearson correlation is $$ {r}_{\max}^2={\left(\frac{COV\left({\widehat{\mathbf{y}}}_p,\overset{\smile }{y}\right)}{\sqrt{\operatorname{var}\left({\widehat{\mathbf{y}}}_p\right)\operatorname{var}\left(\mathbf{y}\right)}}\right)}^2\kern0.5em =\kern1em \left(\frac{\sigma_a^2}{\sigma_a^2+{\sigma}_e^2}\right)={h}^2 $$. However, using the observed *Za* based on full data (*n*) and the predicted BLUPs $$ {\widehat{y}}_{p_{(k)}} $$ based on *n-k* data the $$ {r}_{\max}^2={\left(\frac{COV\left({\widehat{\mathbf{y}}}_p,\mathbf{Z}\mathbf{a}\right)}{\sqrt{\operatorname{var}\left({\widehat{\mathbf{y}}}_p\right)\operatorname{var}\left(\mathbf{Z}\mathbf{a}\right)}}\right)}^2\kern0.5em =\kern1em \left(\frac{G_{12}^2}{G_1{G}_2}\right) $$ where *G*_12_ is the covariance between missing and observed genotypes, *G*_1_ is the covariance among the missing genotypes and *G*_2_ the covariance between the observed genotypes. In this last case, the threshold is equal 1 given that *G*_12_^2^ = *G*_1_*G*_2_. Therefore, the *r*_max_^2^ based on observed and predicted GBVs are re-scaled to a maximal threshold equal to 1.

Using the predicted breeding values we ranked the 10 % of the most resistance lines in order to select the best germplasm. These predicted breeding values include all markers used in this analysis; presenting, therefore, minor and larger associative markers.

## Abbreviations

BSSV, Bayesian stochastic search variable; EM, expectation-maximization; ERIS, ear rot incidence score; G x E, Genotype –By- Environment; GBLUP, genomic best linear unbiased prediction; GBV, genomic breeding value; GS, genomic selection; GWAS, genome-wide association studies; GWS, genome-wide selection; IBS, identity-by-state; MAS, marker-assisted selection; MCMC, Markov Chain Monte Carlo; NESR, number of ears with symptoms of rot; NSSS, non-stiff stalk synthetic; PCA, principal component analysis; PRK, percentage of rotten kernels; QTLs, quantitative trait loci; REML, residual maximum likelihood; rr-BLUP, the ridge regression best linear unbiased prediction; SNPs, single nucleotide polymorphisms; SSS, stiff stalk synthetic

## Additional files

Additional file 1: Figure S1. Running mean derived from 100 sampling of 5-fold cross-validation in rr-BLUP. GBV vs. GBV means prediction based on BLUPs from full dataset *n* (given as true values) and those predicted by *n-k*. GBV vs. *y* means the prediction based on phenotypic values and BLUPs from *n-k* dataset. (PNG 582 kb) Additional file 2: Genome dataset. (TXT 20871 kb) Additional file 3: R-code. (R 4 kb)

## References

[1] Ali ML, Taylor JH, Jie L (2005). Molecular mapping of QTLs for resistance to Gibberella ear rot, in corn, caused by Fusarium graminearum. Genome.

[2] Barton NH, Keightley PD (2002). Understanding quantitative genetic variation. Nat Rev Genet.

[3] Baumann D, Baumann K (2014). Reliable estimation of prediction errors for QSAR models under model uncertainty using double cross-validation. J Cheminform.

[4] Bigirwa G, Kaaya AN, Sseruwu G (2007). Incidence and severity of maize ear rots and factors responsible for their occurrence in Uganda. J Appl Sci.

[5] Brazil. Portaria n. 11 de 12 de abril de 1996. Estabelece critérios complementares para classificação do milho. Diário oficial da União, Brasilia. 1996.

[6] Brito AH, Von Pinho RG, Luiz J (2008). Controle químico da Cercosporiose, Mancha-Branca e dos Grãos Ardidos em milho 1.

[7] Casa RT, Reis EM, Zambolim L (2006). Doenças do milho causadas por fungos do gênero Stenocarpella. Fitopatol Bras.

[8] Casella G, George EI (1992). Explaning the Gibbs Sampler. Am Stat.

[9] Clements MJ, Kleinschmidt CE, Maragos CM (2003). Evaluation of inoculation techniques for Fusarium Ear Rot and Fumonisin contamination of corn. Plant Dis.

[10] Crossa J, Campos GDL, Pérez P (2010). Prediction of genetic values of quantitative traits in plant breeding using pedigree and molecular markers. Genetics.

[11] de Los Campos G, Hickey JM, Pong-Wong R (2013). Whole-genome regression and prediction methods applied to plant and animal breeding. Genetics.

[12] Endelman JB (2011). Ridge regression and other kernels for genomic selection with R package rrBLUP. Plant Genome.

[13] Fernando RL, Garrick DJ (2013). Implementing a QTL detection study (GWAS) using genomic prediction methodology. Methods Mol Biol..

[14] Flint J, Mackay TFC (2009). Genetic architecture of quantitative traits in mice, flies, and humans. Genome Res.

[15] Gianola D (2013). Priors in whole-genome regression: the bayesian alphabet returns. Genetics.

[16] Gianola D, de los Campos G, Hill WG (2009). Additive genetic variability and the Bayesian alphabet. Genetics.

[17] Gianola D, Weigel KA, Krämer N (2014). Enhancing Genome-Enabled Prediction by Bagging Genomic BLUP. PLoS One.

[18] Habier D, Fernando RL, Dekkers JCM (2007). The impact of genetic relationship information on genome-assisted breeding values. Genetics.

[19] Heslot N, Akdemir D, Sorrells ME, Jannink J-L (2014). Integrating environmental covariates and crop modeling into the genomic selection framework to predict genotype by environment interactions. Theor Appl Genet.

[20] Heslot N, Jannink J-L, Sorrells ME. Perspectives for genomic selection applications and research in plants. 2014:1–30. doi: 10.2135/cropsci2014.03.0249

[21] Heslot N, Yang H-P, Sorrells ME, Jannink J-L (2012). Genomic selection in plant breeding: A comparison of models. Crop Sci.

[22] Hoffman GE (2013). Correcting for population structure and kinship using the linear mixed model: theory and extensions. PLoS One.

[23] Lorenzana RE, Bernardo R (2009). Accuracy of genotypic value predictions for marker-based selection in biparental plant populations. Theor Appl Genet.

[24] Mackay TFC (2001). The genetic architecture of quantitative traits. Annu Rev Genet.

[25] Meuwissen THE, Hayes BJ, Goddard ME (2001). Prediction of total genetic value using genome-wide dense marker maps. Genetics.

[26] Odriozola E, Odeón A, Canton G (2005). Diplodia maydis: a cause of death of cattle in Argentina. N Z Vet J.

[27] Patterson HD, Thompson R (1971). Biometrika trust recovery of inter-block information when block sizes are unequal. Biometrika.

[28] Pereira GS, Camargos R Balestre M Von Pinho, RG, Melo WMC Indirect selection for resistance to kernel rot and leaf diseases in maize lines using biplots. Genet Mol Res. 2015. 14:11052-62. doi:10.4238/2015.September.21.18. 10.4238/2015.September.21.18 26400335

[29] Pflieger S, Lefebvre V, Causse M (2001). The candidate gene approach in plant genetics: a review. Mol Breed.

[30] Raman H, Raman R, Kilian A (2014). Genome-wide delineation of natural variation for pod shatter resistance in Brassica napus. PLoS One.

[31] Reid LM, Woldemariam T, Zhu X (2002). Effect of inoculation time and point of entry on disease severity in Fusarium graminearum, Fusarium verticillioides, or Fusarium subglutinans inoculated maize ears. Can J Plant Pathol.

[32] Romay MC, Millard MJ, Glaubitz JC (2013). Comprehensive genotyping of the USA national maize inbred seed bank. Genome Biol.

[33] Rossouw JD, van Rensburg JBJ, van Deventer CS (2002). Breeding for resistance to ear rot of maize, caused by Stenocarpella maydis (Berk) Sutton. 1. Evaluation of selection criteria. S Afr J Plant Soil.

[34] Siqueira S, Barrocas EN, Machado C (2014). Effects of Stenocarpella maydis in seeds and in the initial development of corn. J Seed Sci.

[35] Team R core (2014). R: A language and environment for statistical computing.

[36] Tembo L, Asea G, Gibson PT (2014). Quantitative trait loci for resistance to Stenocarpella maydis and Fusarium graminearum cob rots in Tropical Maize. J Crop Improv.

[37] Vitezica ZG, Varona L, Legarra A (2013). On the additive and dominant variance and covariance of individuals within the genomic selection scope. Genetics.

[38] Wray NR, Yang J, Hayes BJ (2013). Pitfalls of predicting complex traits from SNPs. Nat Rev Genet.

[39] Yi N, George V, Allison DB. Stochastic search variable selection for identifying multiple quantitative trait loci. Genetics. 2003;1138:1129–38. 10.1093/genetics/164.3.1129 PMC1462611 12871920

