# Supplementary figures and images for: Genomic selection to resistance to Stenocarpella maydis in maize lines using DArTseq markers
Source: BMC Genet. 2016 Jun 18;17:86. doi: 10.1186/s12863-016-0392-3 (PMC4912722; doi:10.1186/s12863-016-0392-3)

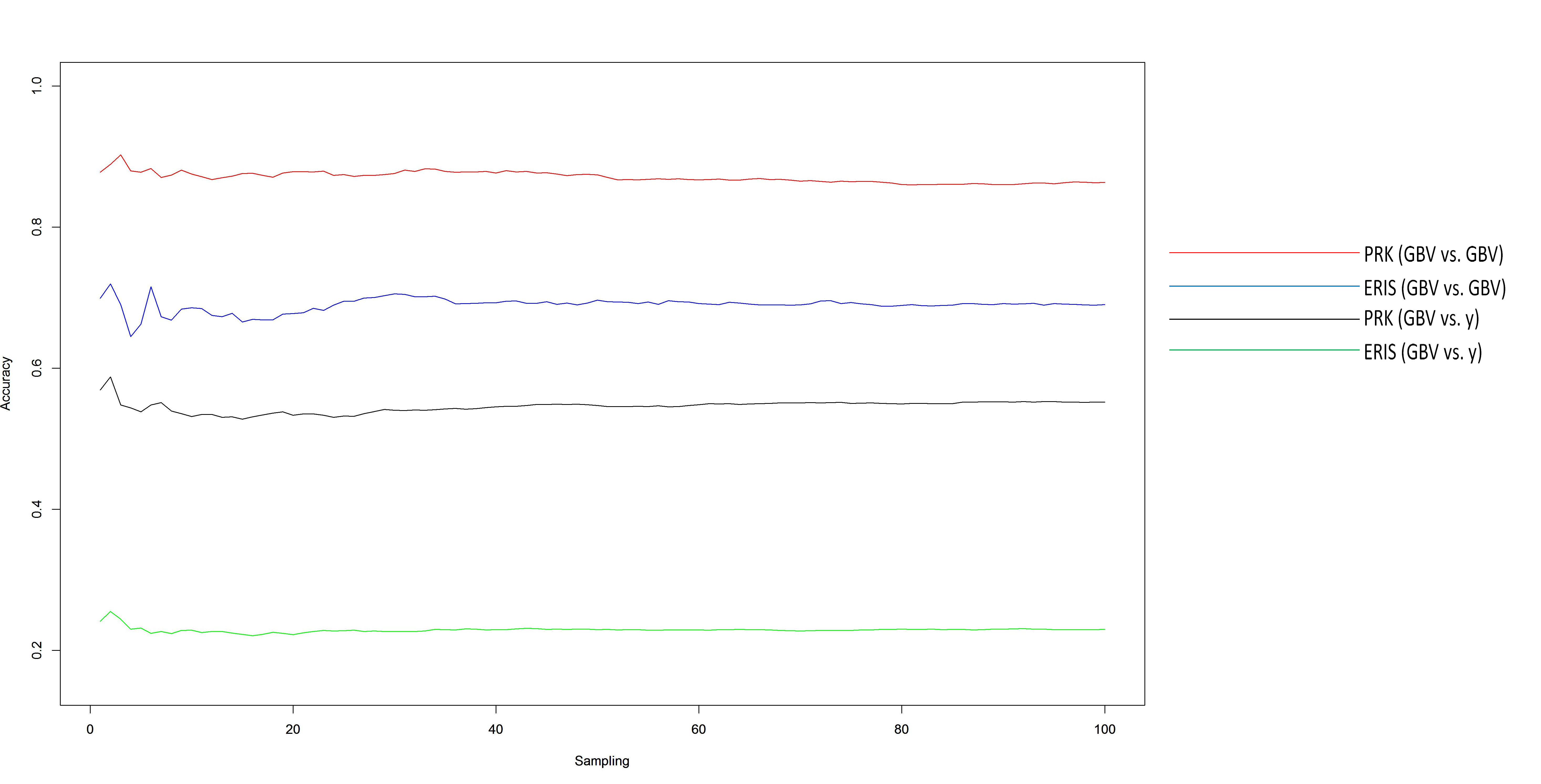

Supplement: Additional file 1: Figure S1. — Running mean derived from 100 sampling of 5-fold cross-validation in rr-BLUP. GBV vs. GBV means prediction based on BLUPs from full dataset n (given as true values) and those predicted by n-k. GBV vs. y means the prediction based on phenotypic values and BLUPs from n-k dataset. (PNG 582 kb) [file 12863_2016_392_MOESM1_ESM.png]
